# Host Genome-Wide Association Study of Infant Susceptibility to *Shigella*-Associated Diarrhea

**DOI:** 10.1128/IAI.00012-21

**Published:** 2021-05-17

**Authors:** Dylan Duchen, Rashidul Haque, Laura Chen, Genevieve Wojcik, Poonum Korpe, Uma Nayak, Alexander J. Mentzer, Beth Kirkpatrick, William A. Petri, Priya Duggal

**Affiliations:** a Department of Epidemiology, Johns Hopkins Bloomberg School of Public Health, Baltimore, Maryland, USA; b International Center for Diarrhoeal Disease Research, Bangladesh, Dhaka, Bangladesh; c Department of Public Health Sciences, Center for Public Health Genomics, University of Virginia School of Medicine, Charlottesville, Virginia, USA; d Wellcome Centre for Human Genetics, University of Oxford, Oxford, United Kingdom; e University of Vermont College of Medicine, Vaccine Testing Center, Burlington, Vermont, USA; f Department of Medicine, Infectious Diseases and International Health, University of Virginia School of Medicine, Charlottesville, Virginia, USA; University of Pennsylvania

**Keywords:** diarrhea, GWAS, *Shigella*

## Abstract

*Shigella* is a leading cause of moderate-to-severe diarrhea globally and the causative agent of shigellosis and bacillary dysentery. Associated with 80 to 165 million cases of diarrhea and >13% of diarrheal deaths, in many regions, *Shigella* exposure is ubiquitous while infection is heterogenous. To characterize host-genetic susceptibility to *Shigella*-associated diarrhea, we performed two independent genome-wide association studies (GWAS) including Bangladeshi infants from the PROVIDE and CBC birth cohorts in Dhaka, Bangladesh. Cases were infants with *Shigella*-associated diarrhea (*n* = 143) and controls were infants with no *Shigella*-associated diarrhea in the first 13 months of life (*n* = 446). *Shigella*-associated diarrhea was identified via quantitative PCR (qPCR) threshold cycle (*C_T_*) distributions for the *ipaH* gene, carried by all four *Shigella* species and enteroinvasive Escherichia coli. Host GWAS were performed under an additive genetic model. A joint analysis identified protective loci on chromosomes 11 (rs582240, within the *KRT18P59* pseudogene; *P* = 6.40 × 10^−8^; odds ratio [OR], 0.43) and 8 (rs12550437, within the lincRNA *RP11-115J16.1*; *P* = 1.49 × 10^−7^; OR, 0.48). Conditional analyses identified two previously suggestive loci, a protective locus on chromosome 7 (rs10266841, within the 3′ untranslated region [UTR] of *CYTH3*; *P*_conditional_ = 1.48 × 10^−7^; OR, 0.44) and a risk-associated locus on chromosome 10 (rs2801847, an intronic variant within *MPP7*; *P*_conditional_ = 8.37 × 10^−8^; OR, 5.51). These loci have all been indirectly linked to bacterial type 3 secretion system (T3SS) activity, its components, and bacterial effectors delivered into host cells. Host genetic factors that may affect bacterial T3SS activity and are associated with the host response to *Shigella*-associated diarrhea may provide insight into vaccine and drug development efforts for *Shigella*-associated diarrheal disease.

## INTRODUCTION

*Shigella*, a genus of Gram-negative rod-shaped bacteria, is the causative agent of shigellosis and the leading cause of bacillary dysentery. *Shigella* species (S. dysenteriae, S. flexneri, S. boydii, and S. sonnei) are the second leading cause of diarrhea-associated mortality globally, accounting for 13.2% of all diarrheal deaths (1, 2). Spread fecal-orally, *Shigella* infection in early childhood is a major cause of moderate-to-severe and bloody diarrhea (3, 4). Low- and middle-income countries (LMIC) are disproportionately affected by diarrheal diseases, with 90% of childhood diarrheal-associated deaths occurring in sub-Saharan Africa and South Asian countries (5). *Shigella* is the second leading cause of watery diarrhea in children under 5 years within LMIC, and these rates likely underestimate the true burden of infection (1, 6). *Shigella* infections contribute to elevated rates of stunting at the population level and increased inflammatory markers, which may have long-term adverse effects on the cellular architecture of gastrointestinal tissues and cognitive development and may affect mucosal vaccine effectiveness (7, 8). While effective therapy involves both prompt oral rehydration and antibiotics, the rising prevalence of drug resistance in high-burden areas suggests prevention through vaccination will be the most effective method of reducing *Shigella*-associated morbidity and mortality globally (2). In many regions, exposure to *Shigella* is ubiquitous, while only a subset of individuals develop symptomatic disease. While risk factors for shigellosis include nonexclusive infant breastfeeding and poor hygiene practices within the household (9, 10), significant heterogeneity in *Shigella*-associated infection remains.

ABO blood group antigens are another potential risk factor, as a study of individuals with blood group B had an increased risk of shigellosis (11). This ABO association suggests an immunogenetic role in host susceptibility to *Shigella*-associated diarrhea and may explain some of the heterogeneity in disease (1, 6). As host genetic factors have been associated with the risk of other diarrhea-associated pathogens, including *Cryptosporidium* and Entamoeba histolytica (12, 13), we aimed to identify the host genetic loci associated with infant susceptibility to *Shigella*-associated diarrhea using two well-characterized birth cohorts from Dhaka, Bangladesh (14, 15). Identifying host factors which influence the immune response to *Shigella* will improve our understanding of the biological mechanisms of *Shigella*-associated diarrhea and may reveal mechanisms of host-pathogen interactions critical for *Shigella* invasion and pathogenesis. Host genetics-focused research can also inform vaccine and drug development efforts, which are needed to curtail the spread or emergence of antibiotic-resistant strains of *Shigella* (2).

## RESULTS

In the PROVIDE and CBC studies, 20% and 31% of infants experienced at least one *Shigella*-associated diarrheal event within their first 13 months of life, respectively. In both cohorts, cases were observed to have had on average more diarrheal events (PROVIDE cases, 4.4; CBC cases, 5.4) than controls (PROVIDE controls, 3.6; CBC controls, 3.3). In cases, the first *Shigella*-associated diarrheal event occurred on average 115 (PROVIDE) to 154 (CBC) days after an infant’s first-ever diarrheal event (Table 1). We observed that the majority of diarrheal events contained multiple pathogens (7, 16), with at least half of identified *Shigella*-associated diarrheal events containing enteroaggregative Escherichia coli (EAEC) at detectable levels. While the Global Enteric Multicenter Study (GEMS) identified EAEC and its virulence factors to be associated with nondysentery moderate-to-severe diarrhea in low- and middle-income countries, including Bangladesh (17), and an increased risk of death in young children (18, 19), the GEMS case-control study included children who reported to sentinel health centers with diarrhea. PROVIDE, a community-based birth cohort, found EAEC to be almost entirely subclinical and *Shigella* to be diarrhea associated (7). Regardless, the carriage of both *Shigella* and EAEC, or the presence of other pathogens, may have potentially impacted our findings. There were no differences in sex, the number of days infants were exclusively breastfed, household size, family income, or family toilet facility between cases and controls (Table 1). CBC infants with *Shigella* were more likely to have lower weight-for-age Z-score (WAZ) values on average than controls at both 1 week (*P* = 0.01) and 1 year of life (*P* = 0.02), but this was not observed in the PROVIDE cohort (*P* > 0.05). In both cohorts, *Shigella* cases had lower height-for-age z-score (HAZ) values at 1 week and 1 year of life than controls; however, this difference was not statistically significant (Table 1). The average severity of diarrheal events (assessed via Ruuska score) was less severe for cases in the PROVIDE cohort, but did not differ between cases and controls in the CBC cohort (20). While an increased risk of shigellosis was described in a small study (*N* = 85) for individuals with the ABO blood group B (*P* < 0.01) (11), this was not observed in the PROVIDE cohort. Rather, slightly more PROVIDE cases had an AB blood group (15%) than controls (9%) (*P* = 0.20) (Table 2). As the distribution of ABO blood groups among PROVIDE’s *Shigella* cases and controls do not differ from 128,506 Bangladeshi blood donors (*P* = 0.32) (21), our findings suggest ABO blood group is not significantly associated with *Shigella* infection risk.

**TABLE 1:** Cohort demographics^*a*^

Characteristic	Value					
	PROVIDE cohort			CBC cohort		
	Cases (*N* = 93)	Controls (*N* = 336)	*P* value	Cases (*N* = 50)	Controls (*N* = 110)	*P* value
Male sex (*N* [%])	52 (56)	179 (53)	0.74	26 (52)	44 (40)	0.21
WAZ (Z-score)						
Enrollment	−1.29	−1.3	0.93	−1.53	−1.1	0.01
1 yr	−1.27	−1.14	0.32	−1.35	-0.92	0.02
HAZ (Z-score)						
Enrollment	−0.91	−0.89	0.83	−0.95	−0.84	0.53
1 yr	−1.6	−1.49	0.49	−1.64	−1.24	0.09
No. of diarrheal events (mean [SE])	4.4 (0.33)	3.6 (0.13)	0.02	5.4 (0.34)	3.3 (0.26)	4.3 × 10^−6^
Age at first diarrheal event (days) (mean [SE])	142 (9.23)	124 (4.53)	0.09	128 (11.88)	130 (8.58)	0.87
Age at first *Shigella*-associated diarrheal event (days) (mean [SE])	257 (8.59)	282 (10.79)				
Days of exclusive breastfeeding (mean [SE])	114 (5.97)	119 (3.32)	0.5	113 (9.31)	119 (6.57)	0.58
Avg Ruuska severity score for diarrheal events (mean [SE])	6.9 (0.21)	7.4 (0.12)	0.02	10.5 (0.29)	10.2 (0.27)	0.41
Shared toilet (*N* [%])	76 (82)	292 (87)	0.27	34 (68)	80 (73)	0.67
No. of individuals in the household (mean [SE])	5.5 (0.3)	5.2 (0.1)	0.42	5.6 (0.3)	5.3 (0.2)	0.56
Avg monthly income (Taka) (mean [SE])	13,000 (1037)	13,000 (518.3)	0.88	18,000 (1838.5)	16,000 (1239.5)	0.49

a The distribution of clinical phenotypes and baseline demographics and clinical measures for the PROVIDE and CBC studies. Continuous variables are presented with their mean (standard error [SE]). Categorical values are presented with their observed frequency and column-wise percentage. 1 Bangladeshi taka = $0.012.

**TABLE 2:** ABO blood group distribution from PROVIDE and 128,506 Bangladeshi blood donors

ABO blood group	Frequency (%)			
	PROVIDE cohort			Bangladesh (*N* = 128,506)^*a*^
	Case (*N* = 93)	Control (*N* = 336)	Overall (*N* = 499)	
A	20	25	24	27
AB	15	9	10	9
B	29	29	28	34
O	28	28	27	30
Missing	7	10	11	

a From Dipta et al. (21).

In the joint unadjusted genetic analysis, two genome-wide significant associations were identified, one on chromosome 11 (rs582240 in the transcribed processed pseudogene *KRT18P59*, minor allele frequency [MAF], 29.3%; odds ratio of the meta-analysis [OR_META_], 0.43; *P* = 6.40 × 10^−8^) and one on chromosome 8 (rs12550437 in the long intergenic noncoding RNA [lincRNA] *RP11-115J16*.*1*, MAF, 38%; OR_META_, 0.48; *P* = 1.49 × 10^−7^). Infants with the T allele at rs582240 in *KRT18P59* were less likely to have a *Shigella*-associated diarrheal event than those with the ancestral C allele. This allele was protective in both cohorts (allelic OR_PROVIDE_, 0.45; OR_CBC_, 0.37; pooled MAF, 29.3%; *P*_META_ = 6.40 × 10^−8^). Under this additive model, the protective effect increased for those carrying two copies of the T allele (OR_PROVIDE_, 0.29; OR_CBC_, 0.09), although only four infants were homozygous for the T allele (PROVIDE cases, *n*_TC_ = 26, *n*_TT_ = 3; CBC cases, *n*_TC_ = 16, *n*_TT_ = 1). The MAF of rs582240 in this study was 29.3% and is consistent with published South Asian population estimates in the 1000 Genomes Project (31%) (see Fig. S4 in the supplemental material) (22).

Similarly, infants with an A allele at rs12550437 in *RP11-115J16.1* were less likely to have a *Shigella*-associated diarrheal event than those with the ancestral G allele. This allele was protective in both cohorts (allelic OR_PROVIDE_, 0.48; OR_CBC_, 0.40; pooled MAF, 38.0%; OR_META_, 0.48; *P*_META_ = 1.49 × 10^−7^). Under an additive model, the protective effect increased for those with two A alleles (OR_PROVIDE_, 0.23; OR_CBC_, 0.15; PROVIDE cases, *n*_AG_ = 35, *n*_AA_ = 6; CBC cases, *n*_AG_ = 19; *n*_AA_ = 3).

**FIG 1:**
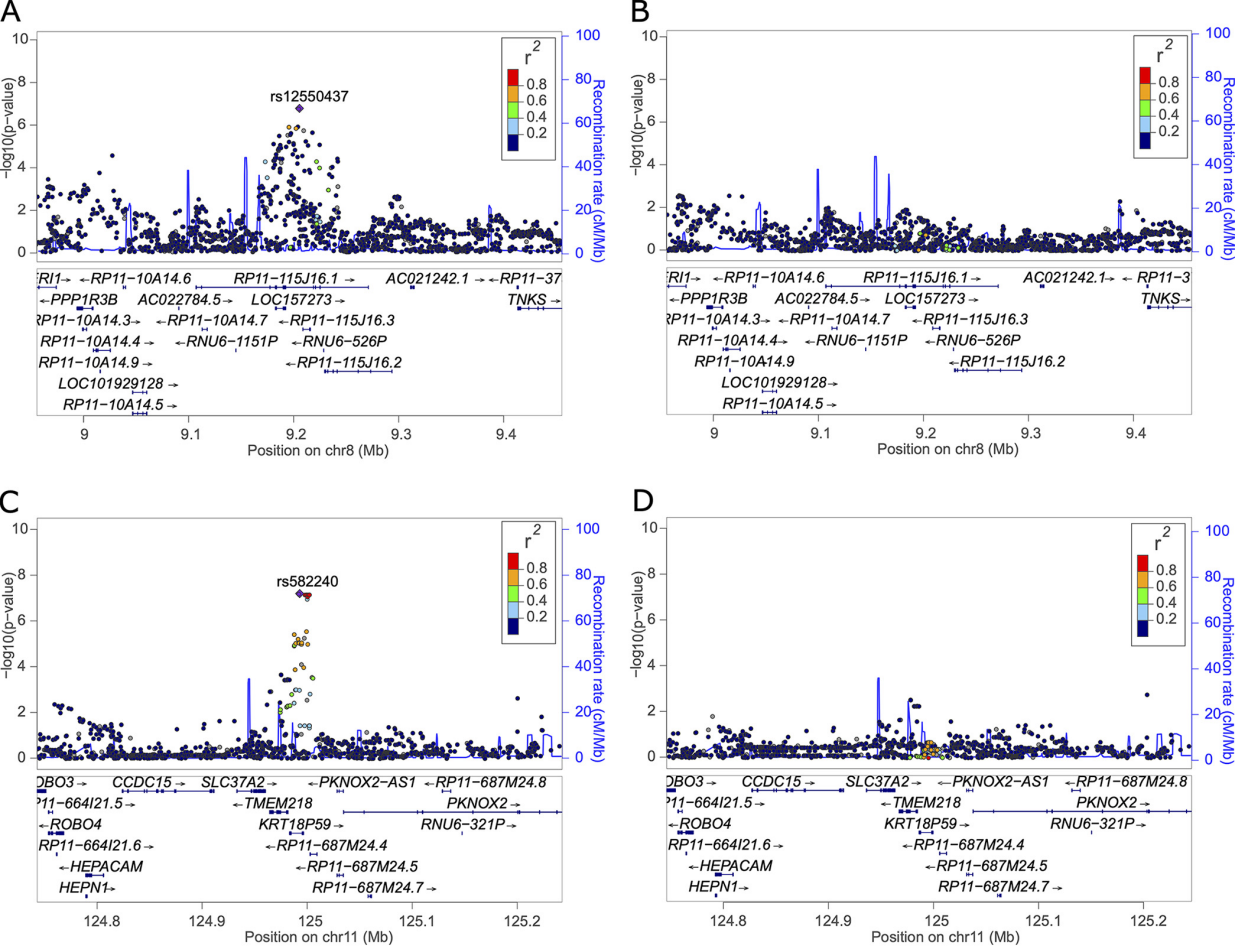
Regional plots of the *RP11-115J16.1* locus on chromosome 8 and pseudogene *KRT18P59* locus on chromosome 11. Regional plots from the unadjusted meta-analyses of the locus surrounding rs12550437 (A) and rs12550437 (C) and meta-analyses conditional on the top SNP identified within the region of interest, i.e., conditioning on rs12550437 (B) or rs582240 (D).

To determine whether these two *Shigella*-associated loci are independent or are each composed of more than a single locus, we performed several conditional analyses. Including the most statistically significantly associated single nucleotide polymorphism (SNP) as a covariate attenuated to the null each locus’s regional signal on chromosomes 8 and 11 and did not alter the effect estimate for the other locus. This suggests the two regions are independent and are each explained by their respective top SNP (Fig. 1).

**FIG 2:**
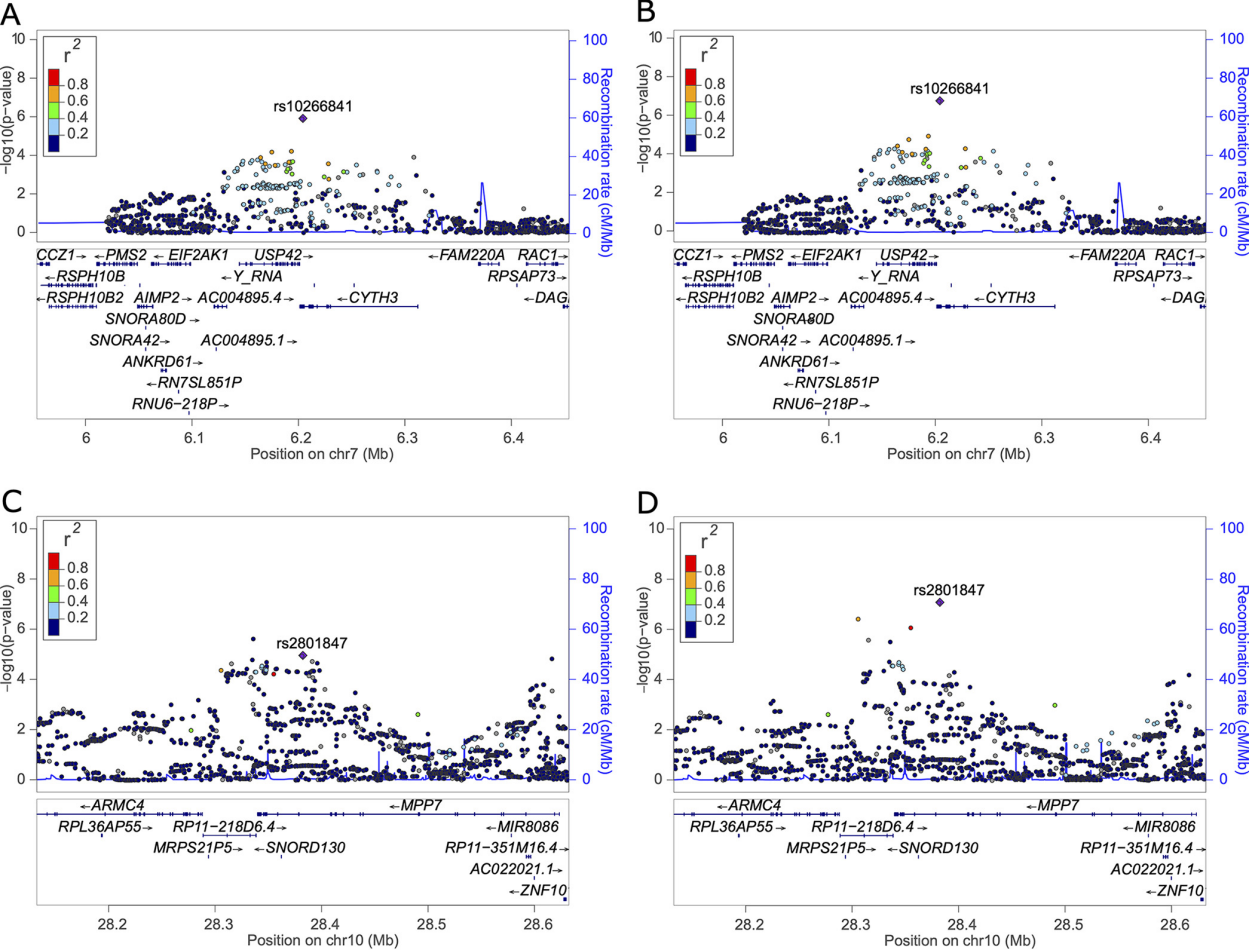
Regional plots of the *CYTH3* locus on chromosome 7 and *MPP7* locus on chromosome 10. Regional plots from the unadjusted meta-analysis for rs10266841 within the 3′ UTR of *CYTH3* (A) and intronic rs2801847 of *MPP7* (C) and meta-analyses conditional on both rs582240 (*KRT18P59*) and rs12550437 (*RP11-115J16.1*) for the *CYTH3* locus (B) and *MPP7* locus (D).

**FIG 3:**
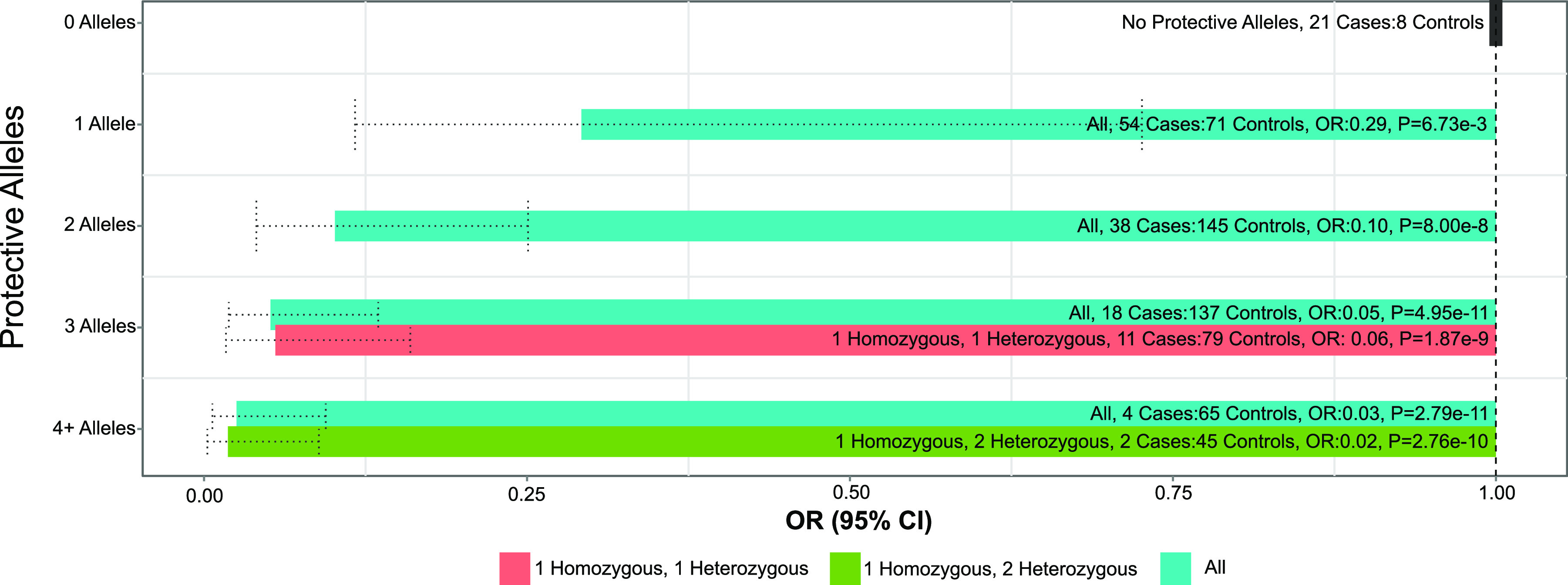
Allelic protective effects across the *KRT18P59*, *RP11-115J16.1*, and *CYTH3* loci. The number of protective alleles across the three identified protective loci, and the associated odds ratios of *Shigella*-associated diarrhea, for infants from the PROVIDE and CBC studies. Estimates of risk and associated statistical significance for each strata of infants are relative to infants homozygous for the ancestral alleles of all three protective loci: *KRT18P59*, *RP11-115J16.1*, and *CYTH3*. “All” reflects the distribution of all protective loci, from 0 to 4 or more protective alleles; “1 homozygous 1 heterozygous,” infants homozygous for 1 protective variant and heterozygous for 1 other; “1 homozygous 2 heterozygous,” infants homozygous for 1 protective variant and heterozygous for the other 2; OR, odds ratio.

Conditioning on these two regions revealed previously suggestive loci on chromosomes 7 and 10, rs10266841 within the 3′ untranslated region (UTR) of *CYTH3* (pooled MAF, 43.4%) and rs2801847 within an intron of *MPP7* (pooled MAF, 7.0%), respectively, to be significantly associated with *Shigella*-associated diarrhea (Fig. 2; see also Fig. S5 and Table S1). Both rs10266841 (3′ UTR of *CYTH3*) and rs2801847 (*MPP7* intron) are predicted to be “possibly damaging” to their respective genes (23, 24). The protective effect associated with rs10266841 (*CYTH3*) became slightly more protective in the conditional meta-analysis (OR, 0.44; P_META_ = 1.48 × 10^−7^) than in the unadjusted meta-analysis (OR, 0.49; *P*_META_ = 1.22 × 10^−6^), while the risk-associated effect of rs2801847 (*MPP7*) became more pronounced in the conditional meta-analysis (OR, 5.51; *P*_META_ = 8.37 × 10^−8^) than in the unadjusted meta-analysis (OR, 3.55; *P*_META_ = 1.11 × 10^−5^) (Table 3). The reduced risk observed among infants with multiple protective alleles in *KRT18P59*, *RP11-115J16.1*, or *CYTH3* matches the expectation for independent and additively protective loci, with infants having a significantly reduced risk of *Shigella*-associated diarrhea for each additional protective allele, regardless of the locus of origin (Fig. 3). For example, infants with a single protective variant from any of these genes were observed to have on average 0.29 (95% confidence interval [CI], 0.12 to 0.73) times the odds of *Shigella*-associated diarrhea compared to those with no protective variants, while those with two or three protective variants (either from the same locus or combined across different protective loci) were observed to have roughly 0.1 (95% CI, 0.04 to 0.25) or 0.05 (95% CI, 0.01 to 0.16) times the odds of *Shigella*-associated diarrhea, respectively (Fig. 3). No additional SNPs reached genome-wide significance after conditioning on all four loci.

**TABLE 3:** Top genome-wide-associated loci from the unadjusted analyses for *Shigella*-associated diarrhea^*a*^

SNP	Ancestral:variant nucleotides	Nearest gene(s)	PROVIDE cohort					CBC cohort					Meta-analysis		
			OR	MAF	No. of cases (*n*_00_, *n*_01_, *n*_11_)	No. of controls (*n*_00_, *n*_01_, *n*_11_)	*P* value	OR	MAF	No. of cases (*n*_00_, *n*_01_, *n*_11_	No. of controls (*n*_00_, *n*_01_, *n*_11_)	*P* value	OR	*P* value	Het. *P* value
rs582240	C:T	*KRT18P59*	0.45	0.29	64, 26, 3	146, 167, 23	3.91 × 10^−5^	0.37	0.31	33, 16, 1	42, 54, 14	5.06 × 10^−4^	0.43	6.40 × 10^−8^	0.69
rs653552	A:G	*RP11-687M24.4*	0.45	0.29	64, 26, 3	146, 167, 23	4.31 × 10^−5^	0.37	0.31	33, 16, 1	42, 54, 14	5.02 × 10^−4^	0.43	6.87 × 10^−8^	0.68
rs147975801	AATAC:A	*KRT18P59*, *RP11-687M24.4*	0.45	0.29	64, 26, 3	146, 167, 23	4.35 × 10^−5^	0.37	0.31	33, 16, 1	42, 54, 14	5.03 × 10^−4^	0.43	6.93 × 10^−8^	0.68
rs12550437	G:A	*RP11-115J16.1*	0.48	0.38	52, 35, 6	116, 165, 56	6.00 × 10^−5^	0.40	0.39	28, 19, 3	34, 52, 24	6.81 × 10^−4^	0.48	1.49 × 10^−7^	0.65
rs10266841	C:G	*CYTH3*	0.46	0.45	42, 44, 7	80, 180, 76	3.26 × 10^−6^	0.68	0.38	23, 22, 5	32, 66, 12	0.10	0.49	1.22 × 10^−6^	0.32
rs2801847	A:G	*MPP7*	2.68	0.07	66, 23, 0	300, 35, 1	5.87 × 10^−5^	2.12	0.07	40, 10, 0	99, 11, 0	0.08	3.55	1.11 × 10^−5^	0.54

a Independent variants, their location, overlapping or nearest genes, genotype frequencies (accounting for dosage), and cohort-specific and meta-analyzed allelic odds ratios and statistical significances are presented. OR, allelic odds ratio; MAF, minor allele frequency; SNP, single nucleotide polymorphism; *n*_00_, number of infants homozygous for the ancestral allele; *n*_01_, number of infants heterozygous for the variant allele; *n*_11_, number of infants homozygous for the variant allele; Het. *P* value, heterozygosity *P* value.

To determine whether the variants significantly associated with infant susceptibility to *Shigella* infection also influence local or distal gene expression, we surveyed the GTEx project for known expression quantitative train loci (eQTLs) (25). Three of the four top region-specific variants (*KRT18P59*, *RP11-115J16.1*, and *CYTH3*) and 13 variants in linkage disequilibrium (LD) (*r*^2^ ≥ 0.8) with these loci are known *cis*-eQTLs or *cis* splice quantitative train loci (*cis*-sQTLs) (see Table S2). It is therefore likely that the top variants in these regions identified in our meta-analysis alter the RNA expression of their overlapping or nearby genes. Limiting our eQTL analysis to the available GTEx gastrointestinal tissues (i.e., stomach, transverse colon, sigmoid colon, and small intestine), the rs582240 (*KRT18P59*), rs12550437 (*RP11-115J16.1*), rs2801847 (*MPP7*), rs10266841 (*CYTH3*), and their nearby variants (*r*^2^ ≥ 0.8) were not associated with the mRNA expression of any gene. However, screening all suggestively associated sites across the genome (*P* ≤ 5 × 10^−5^), which might include additional loci involved in infant susceptibility to *Shigella*-associated diarrhea, for associations with the mRNA expression of nearby genes within these gastrointestinal tissues revealed 73 eQTLs. Seventy of these variants are located across 110 kb on chromosome 10 and were associated with reduced *MASTL* and increased *LINC00202-1* mRNA expression. Several of these same eQTLs were also associated with reduced *YME1L1* expression, a gene adjacent to *MASTL* (see Fig. S6). All genome-wide loci suggestively associated (*P* < 5 × 10^−5^) with *Shigella*-associated diarrhea, with their genomic location and overlapping or nearest gene and its associated function, can be found in Table S3.

## DISCUSSION

The most important result of this work was the discovery of four genetic loci associated with infant susceptibility to shigellosis. Based on the available literature, each of these loci may interact with the *Shigella* type III secretion system (T3SS) or its components. The T3SS, through which *Shigella* injects virulence factors and effector proteins into host epithelial cells, enables *Shigella* to manipulate the transcriptional regulation and chromatin remodeling of host epithelial cells and is required for bacterial invasion and pathogenesis (26, 27).

Our initial joint GWAS identified two genome-wide significant loci to be protective for *Shigella*-associated diarrhea in infants. The first locus, the SNP rs12550437, lies within a lincRNA of unknown function, *RP11-115J16.1*, an alias of the uncharacterized but validated noncoding RNA LOC157273 (Fig. 1). lincRNAs can alter transcriptional regulation and chromatin remodeling, serve as protein/RNA scaffolds, and inhibit protein or mRNA activity (28). Rs12550437 is a *cis*-eQTL associated with the reduced expression of PEAK1 related, kinase-activating pseudokinase 1 (*PRAG1*) within thyroid tissue (*P*_GTEx_ = 1 × 10^−4^) (see Table S2 in the supplemental material). *PRAG1*, or pragmin, is one of few human proteins containing a Glu-Pro-Ile-Tyr-Ala (EPIYA) motif (29), the hallmark of a class of bacterial effector proteins which are introduced into the cell via bacterial secretion systems and manipulate the transcriptional activity or signaling of host cells upon tyrosine phosphorylation, a type of posttranslational modification (30).

*Shigella* harbors a large number of proteins integral to metabolism, T3SS structure, effector function, transcriptional and posttranscriptional regulation, growth, and virulence, which all undergo tyrosine phosphorylation (31). Like bacterial EPIYAs, pragmin undergoes tyrosine phosphorylation at its EPIYA motif and binds and sequesters the Src family tyrosine kinase (SFK) inhibitor Csk to the cytoplasm, preventing it from phosphorylating and inhibiting SFK activity at the plasma membrane and leading to increased membrane-bound SFK activity (29). As active SFKs promote additional pragmin phosphorylation, pragmin-associated reduction of SFK-inhibiting Csk creates a positive feedback loop of SFK activation and pragmin tyrosine phosphorylation (29, 32). SFKs are especially important during *Shigella* infection, as Src is the primary driver of TBK1 autophosphorylation and the activation of type I interferon production while also contributing to the localized recruitment of inflammatory and phagocytizing cells to the site of intracellular infection (33–35). These type I interferons are produced upon bacterial invasion by S. flexneri and are critical modulators of inflammatory processes and initiators of the adaptive immune response (36). SFK activity may also directly contribute to *Shigella* invasion, which requires Src-dependent actin polymerization. Within the context of enriched Src activation, the T3SS component IpaC promotes increased Src-dependent formation of epithelial ruffles and actin-rich structures resembling *Shigella*-specific entry sites (37). As just a small subset of bacterial effectors are sufficient for *Shigella* invasion, without any observable deleterious effects on Src recruitment at bacterial entry loci (31, 37), host-derived mediators of SFK activity therefore play an important role. We observe that rs12550437, a *PRAG1* eQTL, is significantly associated with reduced *Shigella*-associated diarrhea risk. A potential mechanism by which host-derived EPIYA-containing pragmin abundance mediates the risk of *Shigella*-associated pathogenesis is via reduced sequestration of the SFK inhibitor Csk, limiting the abundance or activity of membrane-bound SFKs required for efficient bacterial invasion.

Additional host kinases are required for efficient *Shigella* T3SS-mediated invasion, including serine/threonine kinase 11 (STK11), the tyrosine kinases Abl and Arg, and Bruton’s tyrosine kinase (Btk). STK11 likely mediates *Shigella*’s ability to resolve cellular protrusions into vacuoles, which are engulfed by nearby cells via recruitment of tyrosine kinase receptors and tyrosine phosphorylation within these protrusions (38). The host tyrosine kinases Abl and Arg are activated by *Shigella* infection and recruited to the site of bacterial entry to enable bacterial invasion (39), as is Btk, which phosphorylates the host-derived Wiskott-Aldrich syndrome protein (N-WASP) that forms a complex with the *Shigella* protein IcsA to mediate actin tail formation, *Shigella* motility, and invasion (40). While the importance of host kinases cannot be understated (41), the T3SS and its components or effectors also interact with other host proteins linked to loci identified in this GWAS.

The second SNP, rs582240, lies within *KRT18P59*, keratin 18 pseudogene 59, a processed transcribed pseudogene 84.2% identical to the mRNA of keratin 18 (*KRT18*) (comparing NCBI RefSeqs NG_029288.3 and NM_000224.3). Keratins protect cells from damage and stress while also contributing to the cytoskeletal integrity of epithelial cells (42). While *KRT18* can regulate the expression of host cell-surface proteins known to be targets of various microbial pathogens (43), *KRT18* and the intermediate filament vimentin interact with the *Shigella*-derived T3SS component IpaC and are required for stable docking of S. flexneri’s T3SS to the surface of epithelial cells and efficient T3SS-mediated translocation of bacterial effectors (44).

While most pseudogenes are nonfunctional, some are transcribed, highly active, and enriched for rare variants, suggesting they are under strong purifying selection (45). With proximal regions of open and active chromatin, *KRT18P59* is an actively processed and transcribed pseudogene (45). Transcribed pseudogenes can interact with and alter the normal physiological function of their parental homologous genes in a myriad of ways. For example, the oncogenic BRAF pseudogene, 85.7% homologous to wild-type *BRAF*, does not alter *BRAF* expression but activates the MAP kinase signaling pathway through interactions with wild-type BRAF via its shared CR1 domain (46). Alternatively, the transcribed pseudogene *PTENP1* has a significant effect on the expression of its parental homolog, the *PTEN* tumor suppressor gene, when expressed at just 1% of *PTEN*’s concentration by acting as a “sink” or decoy transcript for microRNAs (miRNAs) (47). Different *PTENP1* isoforms also control *PTEN* expression via recruitment of epigenetic modifications to the *PTEN* promoter or via antisense transcription and direct hybridization to *PTEN* mRNA (48). Pseudogene-mediated interference of parental gene function also occurs via pseudogene-derived small interfering RNA (siRNAs) (49), regulatory interdependence of pseudogene and parental gene expression (50), and the translation of pseudogene-derived peptides (51, 52). Furthermore, as pseudogenes are transcribed into a long noncoding RNA (lncRNA), these transcripts can alter gene function or regulation via any method described for lncRNAs (53). Thus, the *KRT18P59* pseudogene could interfere with normal *KRT18* function and mediate *Shigella*-associated diarrhea risk in several ways. As the significantly identified SNP is a *cis*-eQTL for increased *KRT18P59* expression across multiple tissues, including cultured fibroblasts (*P* = 2.2 × 10^−7^), this SNP might reduce *Shigella*-associated diarrhea risk through inhibiting *KRT18* expression or via interfering with its parental gene’s interaction with *Shigella*-derived IpaC at the epithelial cell surface. It is also possible that regulatory interdependence or the coexpression of *KRT18P59* and *KRT18* is required for efficient *Shigella*-invasion, as the initial characterization of the *KRT18*-T3SS interaction utilized short hairpin RNA (shRNA)-based knockdown experiments which might have reduced both *KRT18* mRNA and *KRT18P59* expression (44). Future functional work is needed to elucidate the precise mechanism by which *KRT18P59* affects *Shigella* pathogenesis.

Conditional analyses performed to ensure that no additional loci neighboring the *KRT18P59* or *RP11-115J16.1* loci were driving their observed associations with *Shigella*-associated diarrhea risk revealed two previously suggested genes as significantly associated with infant *Shigella*-associated diarrhea risk. Both the protective variant rs10266841, within the 3′ UTR of *CYTH3*, cytohesin-3, and the increased-risk-associated variant rs2801847, within an intron of the protein coding gene *MPP7*, membrane palmitoylated protein 7, are predicted to be “possibly damaging” for *CYTH3* and *MPP7* function and are potentially indirectly linked to T3SS activity (23, 24). Widely expressed, *CYTH3* is responsible for regulating protein sorting and membrane trafficking and may regulate ADP-ribosylation factor protein 6 (*ARF6*) and ADP-ribosylation factor protein 1 (*ARF1*) function (54). The ARF6 GTPase is recruited to sites of bacterial entry and activated by the T3SS effector IpgD, which also mediates host actin remodeling (55), and is required for efficient invasion of S. flexneri and is involved in a positive feedback loop amplifying S. flexneri entry into host cells (55). Additionally, *Arf1* has been shown to be targeted by S. flexneri effectors VirA and IpaJ, which inhibit intracellular transport and damage epithelial barrier integrity, promoting *Shigella* invasion (56).

MPP7 is a membrane-associated guanylate kinase (MAGUK) protein family member important for epithelial tight junction formation, with *MPP7* loss significantly impairing both tight junction formation and maintenance (57). A host factor associated with *Salmonella* T3SS activity (58), *MPP7* has not been linked to *Shigella* pathogenesis previously. However, tight junctions are established targets of S. flexneri entry and T3SS-mediated virulence (59, 60). Thus, if the possibly deleterious and risk-associated rs2801847 impairs *MPP7* function, increased or more efficient *Shigella* invasion is reasonable.

The identification of multiple loci, all independently associated with *Shigella* risk, could suggest epistasis between *CYTH3*, *MPP7*, *KRT18P59*, and *RP11-115J16.1* (see Fig. S5). However, the observed additive protective effects of being heterozygous across a combination of these loci are equivalent to the protective effects of those with two protective variants from the same loci (Fig. 3), and given our modest sample size, additional functional work is needed to determine whether these four T3SS-related loci interact to influence infant *Shigella* susceptibility. Interactions between these loci are biologically plausible, with variation within these loci affecting *Shigella*-associated diarrhea risk via altered control of epithelial tight junction formation, tyrosine kinase activity, or cytoskeletal structure within the context of genetic variation that affects *Shigella*’s T3SS-mediated docking at these tight junctions.

The importance of cytoskeletal integrity within the context of *Shigella* infection and pathogen-associated diarrhea in early life is further supported by the identification of 70 *Shigella*-associated variants (*P* ≤ 5 × 10^−5^) across a 110-kb region in chromosome 10 as eQTLs across gastrointestinal tissue types for both *MASTL*, microtubule associated serine/threonine kinase like, a critical regulator of mitosis and genomic stability following DNA damage (61), and the lincRNA *LINC00202-1* (Fig. S6).

Keratins, lincRNAs, and *MASTL* all have functions relating to the cytoskeletal integrity of host cells, suggesting that the pathway by which *Shigella* manipulates the host cytoskeleton may provide drug or vaccine targets given their observed association with the risk of infant *Shigella*-associated diarrhea. Furthermore, the identification of multiple independent variants within genes indirectly linked to *Shigella* T3SS components, especially IpaC, and activity as associated with infant *Shigella*-associated diarrhea risk implies pathogen secretion system-targeted interventions may be especially effective at preventing *Shigella*-associated disease.

*Shigella* and other diarrhea-causing pathogens remain a major source of worldwide morbidity and mortality, especially in resource-limited settings. While this host-focused study is of a modest sample size compared to those of many genome-wide association studies, this study is the first of its kind, provides avenues for future functional work to interrogate *Shigella*-mediated pathogenesis, and offers important knowledge related to the host susceptibility to *Shigella* infection. This study expands the role for keratin pseudogenes but especially highlights the host genes that influence or interact with *Shigella* T3SS components as critical for both susceptibility and protection from infection in young children.

## MATERIALS AND METHODS

### Ethics statement.

This study was approved by the Institutional Review Board (IRB) of the Johns Hopkins Bloomberg School of Public Health, the International Center for Diarrheal Disease Research, Bangladesh IRB, and the University of Virginia IRB. Written consent was obtained from the parents or guardians of all individuals included within this study.

### PROVIDE study.

The Performance of Rotavirus and Oral Polio Vaccines in Developing Countries (PROVIDE) study is a birth cohort of 700 infants and their mothers from the Mirpur area of Dhaka, Bangladesh. The objectives of the PROVIDE study have been described in detail (ClinicalTrials.gov registration NCT01375647) (14). Briefly, 700 infant-mother pairs were recruited between May 2011 and November 2014 and followed for the infant’s first 2 years of life. In-home surveillance of diarrhea occurred biweekly, with diarrhea being defined as having at least three loose stools within a 24-h period. Distinct diarrheal events were defined as occurring at least 3 days apart, with stool specimens taken for each diarrheal episode (14). Clinical and demographic variables were collected at each follow-up visit. Additional neonatal, maternal, household, and socioeconomic measures were recorded at baseline.

### CBC study.

The Cryptosporidiosis and Enteropathogens in Bangladesh Birth Cohort (CBC) study is a prospective longitudinal birth cohort which enrolled infants from Mirpur, Dhaka, and the rural region of Mirzapur, Bangladesh. Stool samples and incident diarrheal samples were collected between July 2014 and June 2017, with additional study information described previously (ClinicalTrials.gov registration NCT02764918) (15). We included 231/250 infants from the Mirpur site that had follow-up for the first 2 years of life (15). Diarrhea was defined as having at least three loose stools occurring within a 24-h period. Baseline and biweekly follow-up demographic, socioeconomic, and clinical measures were collected.

### Phenotype definitions.

Only diarrheal stool samples were included in this analysis. A quantitative PCR (qPCR)-based TaqMan gene expression array card (TAC) was used to quantify a wide variety of microbial pathogens, including *Shigella* (62). The same TAC-based method was utilized for both PROVIDE and CBC to quantify the presence of diarrheal-associated pathogens. The *Shigella*-specific TAC primer targets the *ipaH* gene, carried by all four *Shigella* subtypes and enteroinvasive Escherichia coli (EIEC) (63). While there are multiple *ipaH* genes across the *Shigella* genome and its virulence plasmid, which contains many bacterial effectors required for invasion and pathogenesis, the TAC-based assay targets a plasmid-derived *ipaH* which has been shown to provide similar estimates of *Shigella*-associated diarrhea risk compared to those of other virulence factors (16). The EIEC genome also contains *ipaH*; thus, we cannot rule out the possibility some samples reflect EIEC rather than *Shigella*-associated diarrhea without conducting additional confirmatory microbiological analyses. However, we believe our cases reflect *Shigella*-associated diarrhea rather than EIEC-associated diarrhea. This is based on culture analyses identifying *Shigella* to be much more common than EIEC, EIEC being nondiarrhea associated, and a qPCR validation analysis of TAC-based *ipaH*-positive samples confirming *ipaH*-positive samples as S. flexneri or S. sonnei (4, 16). Phenotype and case definitions were based on the observed distribution of the qPCR cycle threshold (*C_T_*) values for the *Shigella*-specific primers, with a lower *C_T_* value indicating the target as more abundant. Cases were defined as children with at least one diarrheal episode positive for *Shigella* (*C_T_* < 30) within the first 13 months of life. Controls were defined as children who never had an observed episode of *Shigella*-associated diarrhea within their first 13 months of life. PROVIDE cohort’s *C_T_* values show a bimodal distribution (see Fig. S1 in the supplemental material), and previous work supports a *C_T_* threshold of 30, which is more conservative than the threshold used for *Shigella*-associated stool samples globally (*C_T_* < 33.1) (16).

A similar distribution of *Shigella*-specific qPCR *C_T_* values is observed for dysenteric/bloody and watery *Shigella*-associated diarrhea, suggesting that dilute watery *Shigella*-associated diarrheal events would correctly identify cases (16). There were 561 infants from the PROVIDE cohort who had TAC data available from their first 13 months (395 days) of life. Of these, 552 infants had complete (i.e., nonmissing) data for the *Shigella*-specific TAC primers, and 110 were defined as cases (at least one *Shigella*-associated diarrheal event with qPCR *C_T_* of <30); 442 were defined as controls (*C_T_* > 30). There were 210 infants with TAC data available from the Mirpur site of the CBC study who completed 13 months (first 395 days) of follow-up. Of these, 209 had complete data available for *Shigella*-specific TAC primers, and 65 infants were defined as cases (at least one *Shigella*-associated diarrheal event with qPCR *C_T_* of <30); 144 infants were defined as controls (*C_T_* > 30).

Of the 336 infants included in the analysis as controls from the PROVIDE cohort (Table 1), 80 infants had a diarrheal event with missing *IpaH*-specific *C_T_* values (23.8%). However, these infants had an average of 4 additional diarrheal events with *ipaH*-specific *C_T_* values >30. For the 110 identified CBC controls, 7 infants had a diarrheal event with missing *IpaH C_T_* values (6.4%), with an average of 4.6 additional diarrheal events with *IpaH C_T_* values >30. Excluding the limited number of visits with missing *IpaH C_T_* values, the majority of *C_T_* values for the PROVIDE controls (95.9%) and CBC controls (98.1%) were ≥35.

For both PROVIDE and CBC, estimates of infant weight-for-age Z-scores (WAZ) and height-for-age Z-scores (HAZ) were derived by comparing the weight or height of the infants included within each respective study to the sex- and age-standardized World Health Organization (WHO) reference populations in the WHO Anthro software, version 3.0.1. Breastfeeding or the number of days the infant was exclusively breastfed prior to the introduction of top feed or mixed feeding was recorded based on self-reports from the mother. Household demographics, including shared toilet facility with neighboring households, number of residents in the household, and average household income in taka was recorded at enrollment by trained study personnel.

### Genetic quality measures.

For CBC, genotyping was performed using the Illumina Infinium 5 Multi-Ethnic Global Array (MEGA). For PROVIDE, genotyping was performed using the Illumina Expanded Multi-Ethnic Genotyping Array (MEGA^EX^). The genetic data were phased and imputed using SHAPEIT (v2.r790) and IMPUTE (v2.3.2) with the 1000 Genomes Project (1000Genomes) phase 3 data as the reference (64, 65). Genetic variants were excluded if the minor allele frequency (MAF) was <5% (*n* = 3,954,457 PROVIDE and *n* = 4,209,102 CBC), they were not in Hardy-Weinberg equilibrium (HWE) (HWE exact tests with *P* ≤ 1 × 10^−5^, *n* = 93 PROVIDE and *n* = 36 CBC), imputed INFO score was <0.70 (*n* = 15 PROVIDE and *n* = 302 CBC), and variant-level missingness was >5% (*n* = 590,340 PROVIDE and 533,669 CBC). No participants in either cohort were excluded due to missing 5% or more variants. After quality control filtering, 6,247,078 and 6,199,160 independent variants remained from PROVIDE and CBC cohorts, respectively.

Plink (v1.90) was used to combine the chromosome-specific files and perform quality control measures and linkage disequilibrium (LD)-based pruning (66). LD-based pruning iteratively identifies variants within a 50 variant-wide window having an *r*^2^ value greater than 0.2. The resulting set of variants was used to determine population substructure in the following analyses. Principal-component analysis was conducted to identify underlying population stratification using the smartPCA program (EIGENSOFT v13050) (67). No population stratification was identified in either cohort as determined by visual inspection of principal components and quantile-quantile plots from the resulting GWAS detailed below. The lambda inflation factors were 0.99 and 1.00 for PROVIDE and CBC, respectively (Fig. S2) (68).

The KING program (v2.1.4) identified cryptic relatedness within and across the cohorts by estimating the genetic relatedness between infants that can lead to inflated test statistics (69). KING detected three pairs of infants as siblings across the cohorts. Two infants from PROVIDE and one infant from CBC (controls) were excluded from the meta-analysis.

### Genetic association testing.

Association tests under an additive model were conducted using the imputed variants that passed quality control in SNPTEST v2.5.2 (70). Statistical tests of association were performed for each SNP and included WAZ, HAZ, diarrheal severity measured via Ruuska score (20), and sex as covariates. Infant HAZ and WAZ estimates were correlated between enrollment and at 1 year of follow-up; therefore, we only included baseline values as covariates. Despite no evidence of population stratification, sensitivity analyses including the top 3 principal components were also performed.

To account for multiple testing concerns, we used a modified Bonferroni correction-based *P* value threshold of 4.37 × 10^−7^ to identify regions of significance and limited our GWAS to variants with a minor allele frequency of >5%. All noncorrelated variants (pairwise *r*^2^ < 0.05) which passed our other quality control measures were identified in the PROVIDE (*n* = 113,183) and CBC (*n* = 114,302) cohorts and subsequently used to obtain this *P* value threshold (4.37 × 10^−7^), as the Bonferroni correction assumes independence between tests. Linkage disequilibrium-based pruning was performed using Plink (v1.90). This threshold falls within the range of established *P* value thresholds (*P* ≤ 5 × 10^−7^ to *P* ≤ 5 × 10^−8^), which are conservative measures to limit potential of a type 1 error, often at the expense of a type 2 error. Due to allelic correlation, even with increasing numbers of SNPs, there is not necessarily an increased number of independent variants, and thus a baseline threshold can be used across the genome (71–73).

### (i) Meta-analysis.

**FIG 4:**
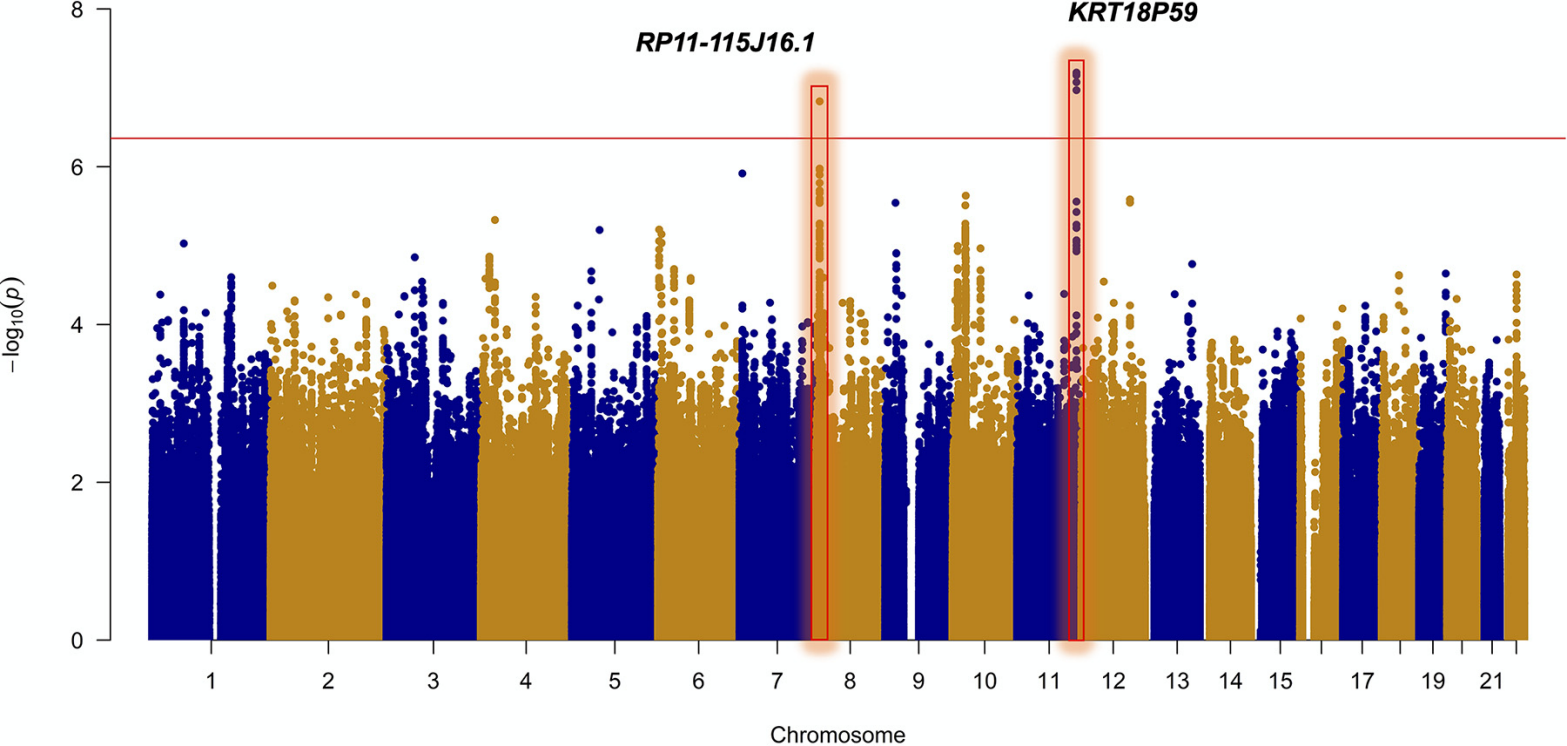
Manhattan plot of the unadjusted joint GWAS. Two genome-wide-associated loci for *Shigella*-associated diarrhea within the first 13 months of life were identified at chromosomes 8 and 11. The red line indicates genome-wide significance (*P* < 4.37 × 10^−7^).

Because of the limited sample sizes of our independent cohorts, the unadjusted analyses were combined in a joint analysis using an inverse-variance method under a fixed-effects additive model in the program META to improve our ability to detect host genetic loci associated with *Shigella*-associated diarrhea (74). Variants with a heterogeneity *P* value of ≤0.05 were removed (*n* = 777,323). The completed meta-analysis included 143 cases, 443 controls, and 5,729,117 variants across the genome. Manhattan plots were generated using the qqman R package for cohort-specific (Fig. S3) and joint analyses (Fig. 4) (68). For each locus of interest, the LocusZoom software tool (v1.4) was used to interrogate regions surrounding the most significantly associated variants for recombination rates (HapMap CEU estimates) and neighboring loci in linkage disequilibrium (75). All variants associated with *Shigella*-associated diarrhea at a *P* value threshold of ≤5 × 10^−5^ were annotated using SNPnexus (76). Functional consequences of genome-wide significant variants and nearby variants in LD (*r*^2^ ≥ 0.8) were assessed using the Ensembl variant effect predictor and LoFtool, a pathogenicity prediction method based on gene-specific ratios of loss of function to synonymous mutations (23, 24).

### (ii) Conditional analysis.

Conditional analyses were performed by including the top associated variant from each locus that reached genome-wide significance (*P* ≤ 4.37 × 10^−7^) as a covariate in a cohort-specific model and then jointly analyzed to determine if the top single nucleotide polymorphism (SNP) explained the associated peak (Fig. 1). If the *P* values of surrounding variants are attenuated after the inclusion of the most significantly associated variant within the model, it indicates the top variant can explain the entirety of the signal observed at this locus. This can be explained by the allelic correlation, or linkage disequilibrium, often observed between SNPs in close proximity. The most significantly associated variant from each locus was chosen, as no variant of known function was observed at either locus.

### Exploration of expression quantitative trait loci.

The variants associated with *Shigella*-associated diarrhea at a *P* value threshold of ≤4.37 × 10^−7^ in the unadjusted or conditional analyses, as well as neighboring variants in LD (*r*^2^ ≥ 0.8), were queried across all Genotype-Tissue Expression (GTEx) tissues in order to identify variants with functional effects related to gene expression. Additionally, all variants associated with *Shigella*-associated diarrhea at a *P* value level of ≤5 × 10^−5^ were included within an analysis restricted to GTEx stomach, colon, and small intestine tissues (25).

### Data availability.

All data are either publicly available from the NIH, via dbGAP, phs001478.v1.p1 (Exploration of the biologic basis for underperformance of oral polio and rotavirus vaccines in Bangladesh) (https://www.ncbi.nlm.nih.gov/projects/gap/cgi-bin/study.cgi?study_id=phs001478.v1.p1), phs001665.v1.p1 (Field studies of cryptosporidiosis and enteropathogens in Bangladesh) (https://www.ncbi.nlm.nih.gov/projects/gap/cgi-bin/study.cgi?study_id=phs001665.v1.p1), or request from the authors.
